# Lectures upon Clinical Materia Medica

**Published:** 1887-12

**Authors:** 


### Lectures upon Clinical Materia Medica. By Prof. E. A. Farrington, M. D. Edited by Dr. Clarence Bartlett. Price, cloth, $6.00; pp. 750. Bœricke & Tafel, 1887.

The death of Dr. Farrington must have been the cause of a common regret to all students of the homœopathic materia medica, for he was one of the best lecturers upon the materia medica our school has known for years. There were faults in Dr. Farrington's teaching, and these are seen in this volume. They were chiefly a decided leaning toward pathological prescribing, and a tendency to a habit of too hasty changing of remedies. Remedies must be carefully chosen, and then allowed to act, if one would gain the best results. These errors being guarded against, one cannot fail to gain valuable help from these lectures. The practitioner, in reading these lectures, must remember that they were prepared for students—beginners in the study of the materia medica—and hence cannot have that completeness and thoroughness of detail which the advanced practitioner needs. Considered as lectures to students, this volume is reliable and complete. Lectures upon drugs are at best but a narrative of the lecturer's experience and a detailing of his views on materia medica. Only in the provings do we get undeniable and reliable *facts*, and in all cases of doubt one should turn to the pathogenesis of a drug. Yet, as one must study drug action from all sides, these clinical lectures are very helpful.

This volume contains lectures upon all the prominent remedies; they are considered by kingdoms. As related drugs are taken up, many valuable comparisons are given.

As Dr. Farrington's lectures are sure to be extensively appreciated, we only add a word of advice: study them thoroughly and carefully; a hasty reading will be useless.

